# Imatinib plasma levels: correlation with clinical benefit in GIST patients

**DOI:** 10.1038/sj.bjc.6605584

**Published:** 2010-02-23

**Authors:** N Widmer, L A Decosterd, C Csajka, M Montemurro, A Haouala, S Leyvraz, T Buclin

**Affiliations:** 1 Division of Clinical Pharmacology and Toxicology, Centre Hospitalier Universitaire Vaudois and University of Lausanne, Hôpital de Beaumont 06.605, Lausanne CH-1011, Switzerland; 2 Multidisciplinary Oncology Centre, Centre Hospitalier Universitaire Vaudois and University of Lausanne, Lausanne CH-1011, Switzerland

**Sir,**

The issue of suitability of therapeutic drug monitoring (TDM) for imatinib continues to fuel controversies. Unlike two previous studies in gastrointestinal stromal tumours (GIST) patients (Judson *et al*, 2005; Delbaldo *et al*, 2006), a recent clinical pharmacokinetic (PK) substudy carried out on the basis of the B2222 trial, evaluating imatinib in patients with unresectable or metastatic CD117-positive GIST, found a correlation between imatinib total exposure and clinical response. Trough levels over 1100 ng ml^−1^ predicted a better overall benefit rate (composite clinical outcome; Demetri *et al*, 2009). These results are thus in line with observations made in CML patients, showing trough levels over 1000 ng ml^−1^ to predict a better molecular response rate (Picard *et al*, 2007; Larson *et al*, 2008).

These concentration–effect relationships confirm and strengthen our results obtained in a population of GIST patients, albeit smaller-sized (38 patients), which we had previously reported in the Journal (Widmer *et al*, 2008). We have indeed observed that higher free imatinib exposure predicts a higher probability of therapeutic response, when taking into account tumour *KIT* genotype. The strongest association was observed in patients harbouring exon 9 mutation or wild-type (*wt*) *KIT*, which is known to decrease tumour sensitivity towards imatinib (Heinrich *et al*, 2003). In fact, we found that, in our population of patients, free plasma concentration (the pharmacologically active species in plasma; i.e., imatinib fraction not bound to *α*_1_-acid glycoprotein (AGP); Widmer *et al*, 2006) was a better predictor of the clinical response rather than total concentration. This free exposure was derived from the total exposure using a mathematical model taking into account the AGP plasma level (Widmer *et al*, 2006). Moreover, we found a significant relationship between this free exposure and clinical response only in patients with exon 9 mutation and *wt KIT*. Of importance, we also observed significant correlations between total, as well as free, imatinib exposure and the occurrence of side effects (Widmer *et al*, 2008).

To better compare our results with those of the B2222 PK substudy, we recomputed maximum a posteriori extrapolations for both total *C*_min_ and free *C*_min_ in our patient samples, rather than considering the global imatinib exposure (area under the plasma concentration–time curve) that was previously analysed (Widmer *et al*, 2008). Among the 38 GIST patients of the previous analysis, AGP plasma levels – required to calculate free *C*_min_ – were available for 36 patients. All these patients were included in an observational study approved by the Ethics Committee of the Lausanne Faculty of Medicine. Informed written consent was obtained from all the participants. A specific population PK model (Widmer *et al*, 2006) was used for this extrapolation (using NONMEM, version VI 2.0, NONMEM Project Group, University of California at San Francisco, San Francisco, CA, USA). We investigated their correlation with clinical benefit, defined as response evaluation criteria in solid tumours (RECIST) complete response, partial response or stable disease, by logistic regression analysis (using Stata version 10.1, Stata Co., College Station, TX, USA).

We found no significant overall association between total *C*_min_ and response in our GIST population. Conversely, imatinib free *C*_min_ was correlated with a clinical benefit, with responders having higher free levels than non-responders (RECIST progressive disease). This relationship did not reach significance over the whole patient sample series (i.e., irrespective of the *KIT* genetic profile; area under the ROC curve=0.594 and *P*=0.26 using logistic regression analysis on log_2_ values of free *C*_min_). However, focusing on exon 9 mutated and *wt KIT* cases allowed the identification of a clear relationship (area under the ROC curve=0.932 and *P*=0.013). The cutoff value of 20 ng ml^−1^ free imatinib plasma trough level corresponded to the best sensitivity (86%) and specificity (100%). The geometric average estimate of imatinib free fraction across our study samples was 1.0% (CV 45%). The mean daily doses of imatinib only tended to be slightly higher in exon 9 or *wt* *KIT* patients compared with exon 11 *KIT* patients (649 mg *vs* 590 mg daily, respectively; *P*=0.07 using *t*-test). Table 1 describes our GIST patient samples and Figure 1 shows the striking difference between the free *C*_min_ values of responder and non-responder exon 9 or *wt KIT* patients (per-sample analysis).

This per-sample analysis was performed because imatinib doses administered to each patient during the course of this 3-year-long observational study could be increased or decreased. The concentration, and possibly the response or adverse events related to treatment, may therefore vary at some point for a given patient. Interestingly, a similar analysis carried out on a per-patient basis (i.e., expressing only a single mean free *C*_min_ and one median response for each individual patient) provided a similar relationship and cut-off, without, however, reaching statistical significance because of the limited number of patients with exon 9 or *wt KIT*. The results from our observational study should therefore still be considered cautiously and will have to be confirmed in a larger patient population. In fact, extrapolation of free concentrations still remains to be formally confirmed by direct measurement of free plasma levels of imatinib in patients’ blood, an aspect that is currently being addressed in ongoing studies initiated at our Institution with GIST and CML patients.

Altogether, the results from the B2222 substudy and our results call for attention to imatinib concentration–effect relationships in the management of GIST patients, which deserve further in-depth exploration. In our opinion, future investigations should take into account not only total plasma concentrations but also free plasma concentrations (either measured or computed with regard to AGP and albumin levels), as well as the tumour genotype. We are currently expanding our set of observations in a larger GIST population in that endeavour. We also agree with the authors of the B2222 PK substudy regarding the urgent need for prospective controlled trials to assess whether a TDM programme would optimise the treatment outcomes in GIST patients, as it has been called for in CML patients (Blasdel *et al*, 2007). Similar considerations might apply as well to other new tyrosine kinase inhibitors currently fleshing out our armament of targeted anticancer agents. Convenient analytical methods already exist for their measurement in patients’ plasma (Haouala *et al*, 2009), but, as for imatinib, further clinical evaluation of their concentration–response relationships and of the benefit of optimising their concentration exposure is warranted in cancer patients.

## Figures and Tables

**Figure 1:**
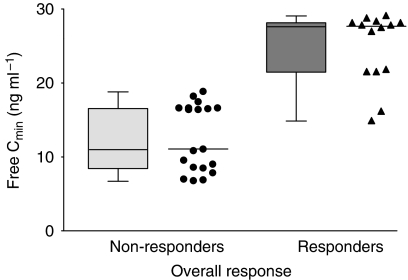
Box plots and individual values of extrapolated free trough concentrations (free *C*_min_) in exon 9 mutated or *wt KIT* patients, contrasting responders’ samples (complete response, partial response, or stable disease; *n*=14; median=25.7 ng ml^−1^) *vs* non-responders’ (progressive disease; *n*=19; median=10.1 ng ml^−1^).

**Table 1:** Left part: description of free trough plasma concentrations (free *C*_min_) deduced from imatinib and AGP levels available in samples from 36 GIST patients; right part: description of the samples from seven patients with exon 9 or *wt* *KIT* identified among 20 with known tumour *KIT* genotype

	**All patients (*wt*, exon 9 and exon 11 *KIT*)**		**Exon 9 and *wt KIT* patients**	
**RECIST response**	***n* (blood samples)**	**Median free *C*_min_ and range (ng ml^−1^)**	***n* (blood samples)**	**Median free *C*_min_ and range (ng ml^−1^)**
Progressive disease	50	13.4 (3.8–22.9)	19	10.1 (6.1–17.4)
Stable disease	63	15.8 (4.5–39.3)	4	19.9 (13.7–20.2)
Partial response	72	13.3 (2.8–33.0)	2	20.5 (14.9–26.1)
Complete response	8	26.0 (25.0–27.0)	8	26.0 (25.0–27.0)

Abbreviations: AGP=α_1_-acid glycoprotein; GIST=gastrointestinal stromal tumour; RECIST, response evaluation criteria in solid tumours.

Each patient provided between 1 and 12 samples over 3 years (median: 4 samples per patient), along with current RECIST response status.
